# The human placenta: new perspectives on its formation and function during early pregnancy

**DOI:** 10.1098/rspb.2023.0191

**Published:** 2023-04-26

**Authors:** Graham J. Burton, Eric Jauniaux

**Affiliations:** ^1^ Department of Physiology, Development and Neuroscience, University of Cambridge, Cambridge, UK; ^2^ EGA Institute for Women's Health, Faculty of Population Health Sciences, University College London, London, UK

**Keywords:** placenta, trophoblast, endometrium, nutrition, development

## Abstract

The placenta has evolved to support the development of the embryo and fetus during the different intrauterine periods of life. By necessity, its development must precede that of the embryo. There is now evidence that during embryogenesis and organogenesis, the development of the human placenta is supported by histotrophic nutrition secreted from endometrial glands rather than maternal blood. These secretions provide a plentiful supply of glucose, lipids, glycoproteins and growth factors that stimulate rapid proliferation and differentiation of the villous trophoblast. Furthermore, evidence from endometrial gland organoids indicates that expression and secretion of these products are upregulated following sequential exposure to oestrogen, progesterone and trophoblastic and decidual hormones, in particular prolactin. Hence, a feed-forward signalling dialogue is proposed among the trophoblast, decidua and glands that enables the placenta to stimulate its own development, independent of that of the embryo. Many common complications of pregnancy represent a spectrum of disorders associated with deficient trophoblast proliferation. Increasing evidence suggests that this spectrum is mirrored by one of impaired decidualization, potentially compromising histotroph secretion through diminished prolactin secretion and reduced gland function. Optimizing endometrial wellbeing prior to conception may therefore help to prevent common pregnancy complications, such as miscarriage, growth restriction and pre-eclampsia.

## 1. Introduction

How the next generation is nourished and supported *in utero* has fascinated philosophers and scientists since at least the days of Aristotle (384–322 BC). The ancient Greeks recognized the central importance of the placenta, the organ that interfaces between the mother and her offspring, although their understanding of its function was far from complete [1]. An issue of importance that was only resolved in the late eighteenth to early nineteenth centuries is that of the separate vascular anatomy of the maternal and fetal placental circulations. In 1754, John Hunter (1728–1793) injected coloured molten wax into the main vessels of the placenta and uterus of dead pregnant women. He demonstrated conclusively that the maternal and fetal blood circulations are separated, thus putting to rest a debate that had engrossed anatomists for many centuries, including Galen, Leonardo da Vinci and Vesalius.

The placenta is unique in many ways. It is the first and the largest fetal organ to develop and during early pregnancy performs the functions of diverse organ systems while these differentiate and mature *in utero*. Furthermore, the placenta undergoes major changes in its own structure and function during gestation while providing constant support for the growing fetus. Immunologically, it is interposed between two genetically related, but different, individuals, the only situation in mammals where this is tolerated [2]. Recent advances in fields as diverse as molecular phylogenetics, genomic imprinting and medical imaging have revolutionized our understanding of the evolution and physiology of the organ, challenging many dogmas held since the nineteenth and twentieth centuries. At the same time, it has become apparent that the influence of the placenta extends beyond the embryonic/fetal period, and that its functions lay the foundation for life-long health of both the offspring and mother [3]. Thus, aberrant placentation not only has immediate consequences for the outcome of a pregnancy, but also predisposes the offspring to metabolic, cardiovascular and neuropsychiatric disorders and certain cancers in adult life due to structural and epigenetic changes in organ systems. These findings have demonstrated that abnormal placentation has its pathophysiological roots in the earliest stages of development from the time of implantation onwards, and possibly, as recent evidence suggests, even in the preparation of the uterus preconceptionally. In this perspective, new insights into the establishment of the human placenta are reviewed, focusing in particular on the first and early second trimesters of pregnancy, and key areas for future research identified.

The placenta displays greater morphological and histological diversity between species than any other organ. These differences may be driven by factors such as the degree of maturation of the offspring required for survival at birth, exposure to pathogens and the risk of vertical transmission to the fetus, the acquisition of ancient transposable elements, and the stability of the environment and habitat which influences maternal food intake and hence the flux of nutrients across the maternal–fetal interface [4–7]. In particular, there are major differences in the degree of invasion of the maternal tissues by the conceptus at the time of implantation. At one extreme is the non-invasive form of placentation, typified by the pig and ruminants, where the conceptus remains within the uterine lumen throughout the pregnancy and there is a simple abutment of the fetal trophoblast, the epithelial covering of the placenta, to the uterine epithelium: the epitheliochorial type placenta [8]. At the opposite extreme is the highly invasive form displayed by the great apes and the human, where the conceptus becomes completely embedded into the uterine wall and erosion into the vasculature allows maternal blood to bathe the trophoblast: the haemochorial placenta. For many years, this diversity was interpreted as an evolutionary progression, with influential authorities considering the human form the most advanced as it permits the earliest and most intimate relationship between the maternal and fetal circulations [9,10]. The assumption was that these features allow for the greatest flux of nutrients across the interface, which enabled the encephalization of the hominins. However, modern molecular phylogenetics have overturned this view, and there is now mounting evidence indicating that the ancestral mammal had an invasive placenta and that the non-invasive epitheliochorial arrangement is a derived state that has arisen by convergent evolution in a number of unrelated orders [11–13]. Avoidance of the complex immunological interactions ensuing from invasive implantation, and their potential involvement in complications of pregnancy, may have been a key selective pressure [14].

Despite these morphological differences, advances over the last 2–3 decades have revealed greater similarities in the development and physiological functioning of the early placenta among the human and other mammalian species than previously appreciated.

## 2. Embryogenesis takes place in a protective low-oxygen environment

One of the most significant advances must be the realization that the haemochorial arrangement is not established in the human until around 13 weeks of pregnancy [15]. Previously, it had been assumed from the presence of maternal erythrocytes within the lacunae, the forerunners of the intervillous space in which the maternal blood later circulates, that an arterial supply is established shortly after implantation, from approximately the fourth week of pregnancy [9,16]. This situation is still depicted in many textbooks of embryology, often with the implication of oxygen exchange despite the fact that the number of erythrocytes observed was surprisingly low and they were pale staining [16]. The change in thinking was stimulated by a combination of findings from different modalities, including Doppler ultrasound imaging and hysteroscopy *in vivo* and perfusion of pregnant hysterectomy specimens *in vitro.* These approaches all failed to detect evidence of significant blood flow in the intervillous space during the first trimester [17–19]. The findings were highly controversial at the time due to arguments over the sensitivity of the imaging equipment and *ex vivo* vasoconstriction of the uterine vasculature [20,21]. Surprisingly, earlier claims that connections between the maternal spiral arteries and the intervillous space could not be seen in placenta-*in-situ* histological material during the first trimester were ignored during the debate [22–24].

**Figure 1.**
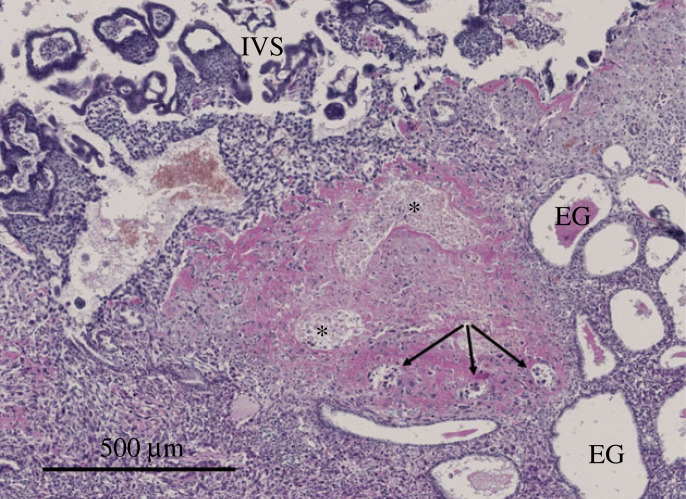
Photomicrograph of the maternal–fetal interface at 55 days of gestation. Aggregates of endovascular trophoblast (asterisks) can be seen occluding the lumen of a spiral artery as it approaches the intervillous space (IVS), preventing free flow of maternal blood into the placenta. A few loosely arranged trophoblast cells can be seen within smaller diameter proximal profiles of the artery (arrows). Note the pink staining fibrin surrounding the artery indicative of physiological remodelling. EG, endometrial glands.

The controversy was resolved by three complementary lines of evidence. First, measurements taken within the placenta with polarographic electrodes revealed a low concentration of oxygen (approx. 25 mmHg) between 7 and 10 weeks of pregnancy that increased 2.2-fold by 11–16 weeks [25–27]. Furthermore, this increase was matched by a rise in expression and activity of the principal antioxidant enzymes within the placental tissues, confirming that it was manifest at the cellular level. Second, an alternative supply of nutrients through histotrophic nutrition from the uterine/endometrial glands during the first trimester was identified [28], as will be discussed later. Third, morphological studies revealed that during most of the first trimester the terminal sections of the maternal spiral arteries leading into the placenta are largely occluded by aggregates of extravillous trophoblast (figure 1) that migrate from the placenta into the lumen of the arteries as part of their physiological remodelling [17,29,30].

The first trimester of human pregnancy corresponds to the periods of embryogenesis and organogenesis, and to appreciate the significance of the low-oxygen environment the requirements of the embryo need to be considered. There is no doubt that the mammalian order evolved from an egg-laying ancestor, and in this context, the chicken is an insightful model. All the nutrients needed to support the chick are contained within the albumin and yolk, and the only additional requirement is a supply of oxygen. Respiratory gas exchange *in ovo* is performed by the highly vascularized chorioallantoic membrane, the equivalent of the mammalian chorioallantoic placenta, which is closely applied to the inner aspect of the shell. However, the chorioallantois only forms around day 10 of incubation, and then rapidly expands to line virtually the entire shell by day 12 [31]. Therefore, for the first 10 days of the normal 21-day incubation period, the supply of oxygen to the embryo is limited to that derived from simple diffusion through the shell pores and the albumin. Consequently, the mean oxygen tensions measured in the region of the head and trunk were 11.4 and 8.3 mmHg (Torr), respectively, on day 4 of incubation, falling to 8.4 mmHg and 6.6 mmHg on day 6 [32]. Furthermore, more than 50% of the measurements were in the range of 0–5 mmHg. It is clear from these studies that embryogenesis can take place in a low-oxygen environment; in fact, there is considerable evidence that such conditions are favourable by reducing the risk of free radical-mediated teratogenesis and maintaining stem cell pluripotency.

Oxygen free radicals are an inevitable by-product of aerobic metabolism and are capable of causing indiscriminate damage to biomolecules and disruption of signalling pathways. Studies have shown that culture under elevated oxygen concentrations or experimental manipulations to generate increased levels of free radicals are associated with elevated risks of a variety of congenital defects [33–35]. Therefore, restricting oxygen availability during the period of organogenesis may reduce the risk of teratogenesis [36]. In the majority of species, the chorioallantoic placenta does not develop and function until organogenesis is nearly complete. For example, the chorioallantoic placentas of the mouse and rat do not function until day 10 of the 21-day pregnancies, and in the horse, the conceptus does not attach to the wall of the uterus until at least five weeks of pregnancy [8]. Thus, in these examples of invasive and non-invasive placentation, respectively, the oxygen supply to the embryo during early pregnancy will be limited to diffusion through the gestational sac. Although oxygen measurements within the sacs are not available for these species, it is likely that equivalent low-oxygen conditions prevail.

Another potential benefit of the low-oxygen conditions is maintenance of the pluripotency of stem cells. The oxygen concentration present in most adult stem cell niches is in the range of 2–8% oxygen, which is consistent with the approximately 2.5% reported in the embryonic fluids during the first trimester [27,37]. Both stem cells and embryonic tissues rely heavily on glycolysis for energy production. To maintain glycolysis and the generation of two moles of ATP per mole of glucose, it is necessary to continually regenerate NAD^+^. In the adult, this is achieved under aerobic conditions through the citric acid cycle, or, if oxygen availability is limiting, by fermentation of pyruvate to lactate. Levels of lactate in the human gestational sac are elevated (0.6 mmol l^−1^) but are not excessively high during the first trimester [27,38], indicating limited reliance on this pathway. Instead, concentrations of polyols, such as ribitol, erythritol and sorbitol, are very high [39]. Formation of these sugar alcohols provides another means of regeneration of NAD^+^ and NADP^+^, and represents some of the oldest carbohydrate metabolic pathways phylogenetically that supported life when it first evolved in an atmosphere devoid of oxygen [40]. Ribitol is formed through an off-shoot of the pentose phosphate pathway, which is highly active in embryonic tissues as it is involved in the formation of nucleic acids and cell proliferation, as well as generating reduced glutathione and boosting antioxidant defences. Polyols are incapable of crossing cell membranes and so act as powerful osmolytes, helping to draw fluid across the maternal–fetal interface and to expand the gestational sac.

Because of the high level of glycolytic activity the ATP/ADP ratio in first trimester placental tissues is the same as that during the second trimester and at term when the prevailing oxygen concentrations are higher and oxidative phosphorylation is more active [41]. Thus, there is no evidence that the tissues are energetically compromised; indeed, this would be inconsistent with their high rates of proliferation and secretory activity. It is therefore inappropriate to refer to the first trimester intrauterine environment as hypoxic, for this implies a condition in which energy demanding activities such as biosynthesis and proliferation are downregulated to aid cell survival [42]. Instead, it should be considered a physiological low-oxygen state. The one thing that is required is a prolific supply of glucose [43], and as will be seen this is provided by the uterine glands in the form of histotrophic nutrition.

## 3. Histotrophic nutrition during embryogenesis

In all species, initial nutritional support for the fertilized ovum and later the blastocyst is provided by the oviductal fluid secreted by the cells lining the Fallopian tube. Once the conceptus enters the uterus, the uterine or endometrial glands take over this role. Histotroph is the generic term used to describe this mix of cellular secretions, cell debris and transudation that is released into the space between the maternal and fetal surfaces and is phagocytosed by the trophoblast [8]. In the past, uterine histotroph was often referred to as ‘uterine milk’, in part due to its high lipid content and in part due to the fact that the ancients believed the fetus suckled on uterine paps in an analogous fashion to the neonates feeding from the breast. Walter Needham (1631–1691) is widely credited with the discovery of ‘uterine milk’, although it is clear from the writings of William Harvey (1578–1657) that he was familiar with the secretions a century earlier [44]. Reliance on histotrophic nutrition continues until the vascularized placenta is established, when it is generally overtaken by haemotrophic exchange between the maternal and fetal circulations. In many species, the two pathways may operate side-by-side for much of gestation, each conveying specific nutrients. Thus, in those with epitheliochorial placentas domed specializations of the trophoblast, referred to as areolae, develop opposite the mouths of clusters of uterine glands [8,45]. Early immunohistochemical studies revealed that at these sites the gland epithelial cells stain intensely for iron during pregnancy, but not in the non-pregnant state [46]. The secretions of the glands and the columnar trophoblast cells opposite also stain positively, but less intensely, suggesting this is an important route for maternal–fetal transfer of iron. The identification of the carrier protein uteroferrin that is induced in the gland cells by progesterone confirmed this assumption, and highlighted the importance of histotroph for embryonic development [47].

**Figure 2.**
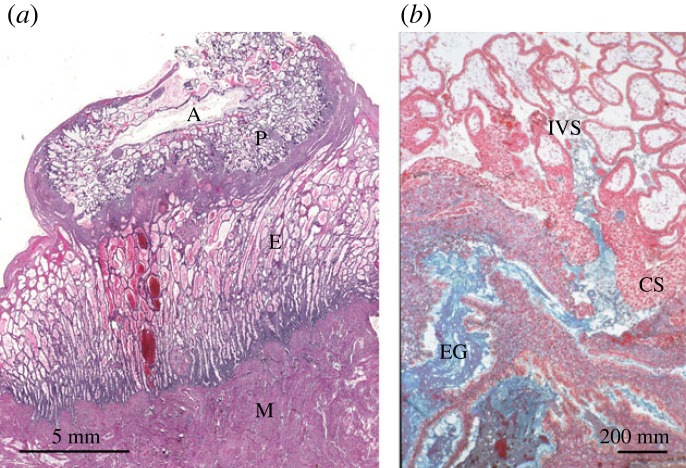
Photomicrograph of an implantation site at 42 weeks of gestation showing histotroph entering the placenta. (*a*) The endometrium (E) beneath the developing placenta (P) is still 6–7 mm thick and contains highly active glands. A, amniotic cavity; M, myometrium. (*b*) An endometrial gland (EG) can be seen discharging its secretions into the intervillous space (IVS) of the placenta through an opening in the cytotrophoblastic shell (CS) that lies at the maternal–fetal interface. Note the heterogeneous nature of the gland secretions, with lipid droplets staining red and carbohydrates blue. Adapted from [28] with permission.

In the human, the histotrophic phase has traditionally been considered very short at approximately two weeks, for it was assumed that the trophoblast cannot access the secretions present within the uterine lumen once the uniquely invasive form of implantation is complete. At the same time, invasion of the maternal vasculature was thought to lead to a precociously early onset of haemotrophic exchange [9,10]. Both these assumptions have subsequently been proved incorrect. Thus, it was demonstrated from placenta-*in-situ* specimens (figure 2) that as the trophoblastic cells of primary anchoring villi migrate into the decidua they break into the underlying uterine glands, allowing their secretions to be discharged into the intervillous space through openings in the developing basal plate [28,48]. Furthermore, maternal glycoproteins secreted exclusively by the glands, such as glycodelin-A and MUC-1, have been shown by immunohistochemistry to be phagocytosed from the intervillous space by the syncytiotrophoblast, where they come to co-localize with the lysosomal pathway [28,49]. The latter finding suggests a proportion may be broken down and used in metabolic and anabolic pathways, in a similar fashion to uptake and breakdown of maternal proteins by the rat yolk sac. It has been estimated that during organogenesis approximately 95% of amino acids in the rat embryo are derived from digestion of maternal proteins rather than transfer of free amino acids from the maternal serum [50]. No data on uptake kinetics or subsequent breakdown and recycling of elements are available as yet for the human, but this would be an interesting area for future research.

A proportion, as yet unknown, of the maternal proteins evade the lysosomes and cross the placenta intact, accumulating in the coelomic and amniotic fluids [51,52]. Despite their maternal origin, these intact proteins do not appear to induce any immunological reaction, most likely because macrophages are the only fetal immune cells present within the villous cores and they do not express HLA-DR [53]. Indeed, the fetal macrophages are also immunopositive for glycodelin, indicating that they too may phagocytose the free protein that has crossed the syncytiotrophoblast [28].

Although long assumed to be a source of nutrition for the embryo, the critical importance of the uterine secretions for development was only recently demonstrated experimentally by suppressing development of the uterine glands in both neonatal female sheep and mice [54,55] . The females still ovulate and can be mated but depending on the degree of suppression of the glands the conceptuses either fail to survive when they enter the uterus or are extremely growth restricted.

Several recent advances have made histotrophic nutrition an important and exciting area for further research. Firstly, evidence from domestic species indicates that during early pregnancy hormones and cytokines secreted from the placental trophoblast upregulate activity of the glands, increasing the release of glycoproteins, growth factors and nutrients [56]. This signalling loop provides a novel mechanism by which the placenta is able to stimulate its own development independent of the fetus, which is of course necessary if the placenta is to support the latter. Circumstantial evidence that a similar mechanism may operate in the human comes from the observation that the epithelial lining of the glands undergoes a characteristic hypersecretory phenotypic change during early pregnancy, known as the Arias-Stella reaction [57]. This response is independent of the site of implantation, suggesting it is mediated by endocrine cues rather than through cell-mediated interactions. Notably, glycogen accumulates in the apical cytoplasm of the columnar cells, and may be released by either apocrine secretion [58] or conversion into glucose through the actions of glycogen phosphorylase [59]. Either way, glycogen rosettes are abundant in the syncytiotrophoblast of villi facing the openings of the uterine glands [28,60], where they presumably fuel the high rate of glycolysis discussed earlier [61].

A second recent advance that has opened new possibilities to investigate both the origin and function of histotroph in the human is the derivation of organoids from the endometrial glands [62,63]. Despite the intriguing Arias-Stella reaction, experimental evidence of a placental–endometrial signalling dialogue in the human has been impossible to obtain due to the inaccessibility of the tissues during early pregnancy. Equally, it has been impossible to characterize the secretome of the glands following implantation, although it is known that the epithelial cells are immunopositive for a number of mitogenic growth factors, including epidermal growth factor, vascular endothelial growth factor and leukaemia inhibitory factor during the first trimester [49]. These factors stimulate the proliferation of cytotrophoblast cells when applied to early villous explant cultures [64], consistent with the concept of a feed-forward system. The derivation of organoids that faithfully replicate the transcriptome of the glands and are genetically stable over many passages *in vitro* is enabling these questions to now be addressed. For example, sequential treatment of the organoids with oestrogen and progesterone upregulates secretion of glycodelin-A (officially referred to as progestagen-associated endometrial protein) and osteopontin (officially referred to as secreted phosphoprotein 1), two of the principal components of the histotroph. This effect is further enhanced by the addition of human chorionic gonadotropin (hCG), placental lactogen and prolactin [62]. A proteomic assessment of the secretome and metabolome performed on fluid aspirated from the central cavity of organoids derived from three donors confirmed a high level (35%) of metabolites pertaining to glucose and glycogen metabolism [65], consistent with the reliance of the placenta on glycolysis described previously. Lipids and amino acids were also identified, along with NAD^+^ and xenobiotics, but analyses for extracellular vesicles, microRNAs and other potential maternal–fetal cross-talk mediators were not performed. Donor-specific variations were found and so larger studies involving more individuals are required to define the range of the normal secretome.

The inside-out orientation of the organoids, with the apical surface of the gland cells facing into a central cavity, is obviously a limitation when it comes to analysis of the secretome. To reverse the polarity, organoids have been successfully seeded onto a three-dimensional collagen scaffold, with and without an underlying layer of decidualized stromal cells, where they generate a confluent epithelial monolayer attached through the basal surface [66]. The gland cells remain responsive to hormonal stimulation, and the large surface area created means that conditioned media could easily be obtained for proteomic, lipidomic and other analyses. The use of chemically defined hydrogels that can be tailored to the needs of individual tissues offers further possibilities by reducing batch variability, and they have already been used successfully with endometrial gland organoids [67]. These pilot experiments show considerable potential and could be developed in the future by the inclusion of maternal immune cells and extravillous trophoblast to bioengineer a more complex and accurate artificial endometrium. Such advances open opportunities for co-culture experiments with either human trophoblast organoids [68,69] or blastoids [70,71] to explore critical events during implantation and establishment of the placenta.

A third advance pertaining to the importance of histotroph in the human was the realization that the pattern of glycosylation of the secretions changes in early pregnancy compared to the secretory phase of the menstrual cycle. During early pregnancy, there is a global reduction in terminal sialylation [72], which may facilitate the absorption of histotroph by the trophoblast but more importantly may protect the mother against unwanted effects of the growth factors secreted by the glands. Although their primary target is the placenta, the growth factors will enter the maternal circulation as a component of the fluid draining from the intervillous space into the uterine veins. A build-up of concentrations in the maternal circulation would place the mother's tissues at risk of excessive proliferation and tumorigenesis. However, the loss of terminal sialylation will ensure any secreted glycoproteins are removed in one circulatory pass by asialylglycoprotein receptors in her liver [73,74], maintaining concentrations in maternal blood at very low levels.

The combination of the trophoblast's ability to upregulate gland function and the change in glycosylation during early pregnancy provides a unique and powerful mechanism to autostimulate placental development in a safe manner. The clinical implications are that the endometrium plays a much more important role in promoting establishment of the placenta and pregnancy than previously recognized, as will be discussed later.

## 4. The role of the secondary yolk sac

While growth factors contained within the histotroph may operate locally on receptors on the placental cells, nutrients need to be transported to the embryo. How is this achieved before the fetal circulation to the placenta is established at approximately 8–10 weeks of gestation? The yolk sac is phylogenetically the oldest of the extra-embryonic membranes and the first to be vascularized through the vitelline circulation [8]. Many species pass through a transient choriovitelline phase of placentation when the yolk sac makes contact with the trophoblast layer and exchange takes place into the vitelline vessels. This mechanism supports the embryo until the definitive chorioallantoic placenta develops.

**Figure 3.**
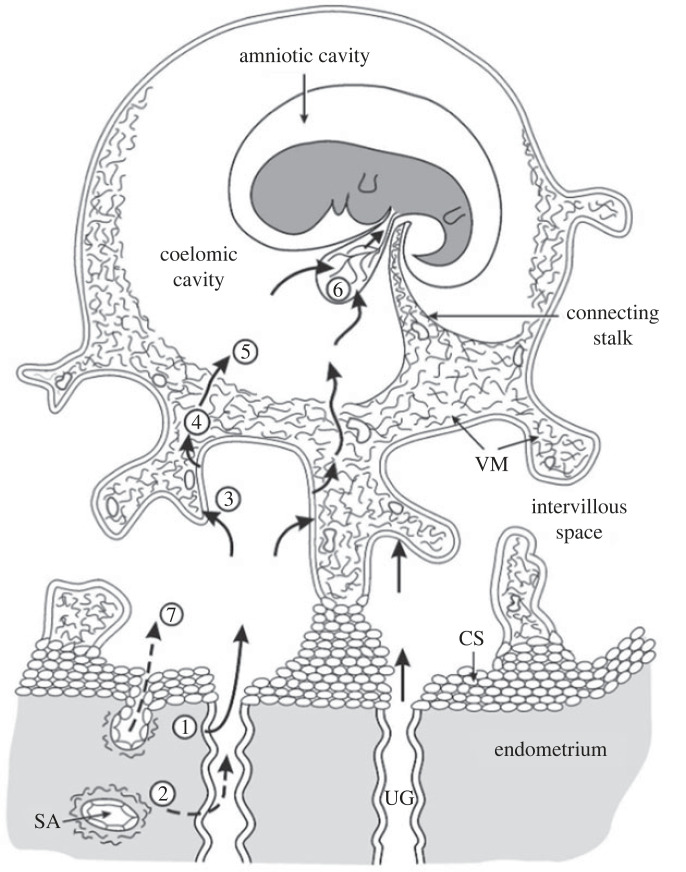
Schematic of the steps along the histotrophic pathway during early human pregnancy. (1) Secretions from the endometrial glands may be supplemented (2, 7) by maternal plasma from the spiral arteries (SA) in the superficial endometrium. Nutrients are taken up by the trophoblast (3), where they may be digested and used in local synthetic pathways, or (4) passed intact into the villous mesenchymal core (VM). The products diffuse into the coelomic fluid (5), from where they may be absorbed by the epithelia of the yolk sac (6) and passed to the fetus through the vitelline circulation. From [80] with permission.

In the human, a choriovitelline placenta never forms morphologically as the secondary yolk sac is small and does not make contact with the inner surface of the trophoblast. Instead, it is separated by a fluid-filled cavity, the extra-embryonic coelom or chorionic cavity, and consequently the human yolk sac is widely regarded as vestigial and of no significant functional capacity in terms of maternal–fetal exchange. This assumption ignores the fact that the secondary yolk sac is the first site of haematopoiesis in the conceptus and displays a rich capillary plexus located under the mesothelial epithelium that faces the coelom [75]. The epithelium also displays all the morphological features of an absorptive surface, and the coelomic fluid that bathes it is rich in amino acids and other nutrients [52]. Recent transcript data from first trimester human yolk sacs have shown a myriad of transcripts encoding transporter proteins, ranging from amino acid transporters through metals and vitamins to inorganic and organic ions [76]. The fact that the majority of these transcripts are conserved across the mouse and chicken yolk sacs, for which there is incontrovertible experimental evidence of their role in nutrient uptake, and that many of the ligands are present in the coelomic fluid strongly suggest that the yolk sac plays an important role in the first weeks of development before the fetal circulation to the placenta is established. In particular, many of the transcripts relate to cholesterol and lipid handling, which are key for cell proliferation and signalling [77]. Unfortunately, data on uptake kinetics and transfer are not available, and it is impossible to confirm a role for the yolk sac *in vivo*. Morphological abnormalities have been reported in 70% of cases of spontaneous miscarriage, but separating cause from effect is not possible [78,79]. Nonetheless, there is much evidence to suggest that the trophoblast, the coelom and the secondary yolk sac combine to act as physiological, rather than a morphological, choriovitelline placenta during the first weeks post-implantation (figure 3) [76].

## 5. The full establishment of the maternal arterial supply to the placenta

Moving echoes seen with Doppler ultrasound and indicative of significant flow become detectable in the placental intervillous space at the start of the second trimester of pregnancy, at around 12–13 weeks [15]. At the same stage of pregnancy, the oxygen concentration within the placenta rises to approximately 60 mmHg [25–27]. This rise corresponds precisely with the end of the period of organogenesis when the risk of free radical-mediated teratogenesis falls sharply. However, the rise still poses an oxidative challenge to the placental tissues, and transient swelling of the mitochondrial intracristal space is observed within the syncytiotrophoblast [26].

It is impossible at present to estimate how rapidly the oxygen concentration increases within an individual placenta, as longitudinal studies with polarographic electrodes cannot be performed for ethical reasons. There is ultrasound evidence, however, that onset of the maternal circulation is coordinated temporo-spatially, starting preferentially in the periphery of the placenta where the extravillous trophoblast aggregates within the spiral arteries are least extensive [81,82]. Placental villi sampled from this region show high levels of oxidative stress and activation of the apoptotic cascade compared to those from the central region of the placenta. These findings have led to the suggestion that the pattern of onset of the arterial inflow plays a role in placental morphogenesis, stimulating regression of villi in the peripheral region of the early placenta to leave the definitive discoid placenta [82,83]. This concept is impossible to test experimentally, but placental shape does appear to be determined by the start of the second trimester [84].

How the onset of the maternal circulation is regulated is not fully understood. As described earlier, during much of the first trimester the terminal portions of the spiral arteries that ultimately supply the placenta are virtually occluded by aggregates of extravillous trophoblast cells that migrate from the placenta. These cells are involved in the physiological remodelling of the spiral arteries, a process that entails loss of the smooth muscle within the wall of the artery and its replacement by fibrinoid material. As a result, these normally highly vasoreactive arteries are converted into flaccid conduits that dilate as they approach the placenta [30]. These changes ensure that the velocity and pressure of the maternal inflow are reduced to levels that avoid damage to the delicate placental villous trees, and that the arteries are unable to constrict and potentially compromise blood supply to the placenta [85]. The cells of the aggregates are only loosely packed together, attached to neighbours by occasional desmosomal junctions [86]. Consequently, a network of intercellular spaces exists that allows plasma to filter through, filling the intervillous space with a clear fluid, but traps maternal erythrocytes [17]. The slow flow of plasma will convey dissolved oxygen to the placental tissues and augment the supply of nutrients derived from the histotroph. Larger channels begin to develop within the trophoblast aggregates from seven weeks of gestation onwards [29,86], and were initially thought to mediate onset of the arterial blood flow. However, their presence does not correlate with an increase in flux through the intervillous space measured with contrast-enhanced ultrasound, which rises sharply at around 13 weeks coincident with the rise in intraplacental oxygen concentration [86]. Recent morphological analyses of vessel diameters indicate that changes in the more proximal radial and arcuate arteries correlate with the increase in flow [87], though how these changes are mediated is uncertain at present.

Nonetheless, there appears to be an association between the timing of onset and the extent of the extravillous aggregates, both in normal pregnancies as described above and in pathological cases. Thus in cases of spontaneous miscarriage onset is both precocious and disorganized, occurring throughout the placenta rather than following a centripetal pattern and causing overwhelming oxidative stress [82,88]. In 70% of these cases, formation of the cytotrophoblastic shell at the maternal–fetal interface from which the arterial ‘plugs’ extend is impaired [89].

## 6. A new paradigm for human early placental development

It is clear from the various strands of evidence presented here that it is necessary to rethink the early development and physiology of the human placenta. Molecular phylogenetics have revealed that the idea the uniquely invasive form of implantation displayed by the human and great apes represents an advanced state of evolution is no longer tenable. Equally, we now appreciate that the period of histotrophic nutrition, initially considered brief, extends for much of the first trimester, providing a rich supply of glucose and lipids whilst maintaining a low-oxygen environment. This process is pivotal to reduce the risk of radical-mediated teratogenesis during embryogenesis and organogenesis.

Phylogenetically old carbohydrate metabolic pathways enable the placental tissues to maintain high energy levels and cell proliferation rates despite the low oxygen tension. Rapid proliferation of the trophoblast is essential to anchor the conceptus in the uterine wall, and to form a thick cytotrophoblastic shell that encapsulates the conceptus and walls-off the maternal spiral arteries while they undergo remodelling to provide a high volume of flow at a low velocity and pressure later in pregnancy. Proliferation is promoted by growth factors contained in the histotroph, and a change in their pattern of glycosylation protects the mother's tissues from undue mitogenic stimulation. In this way, histotroph creates a unique microenvironment that supports rapid growth of the placental tissues safely within the confines of the organ, an arrangement that cannot be achieved by any other means.

**Figure 4.**
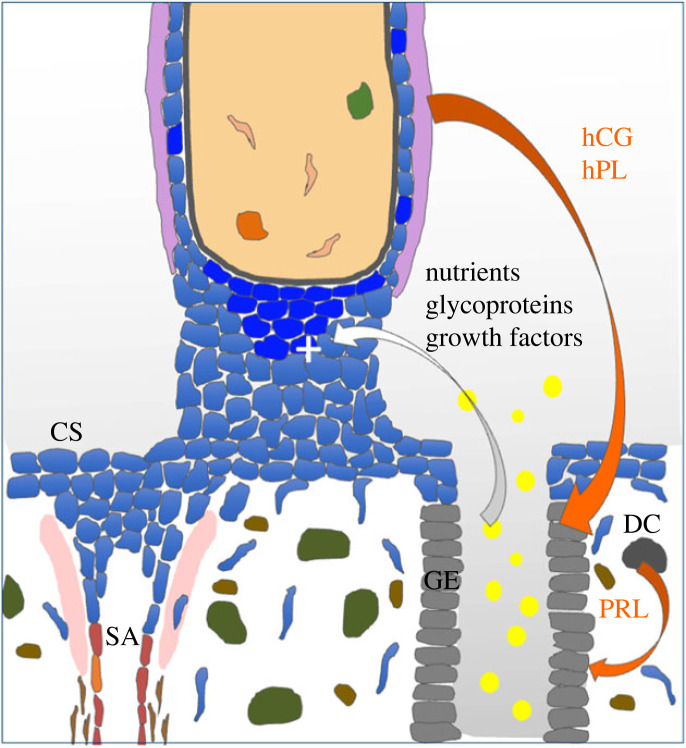
Schematic of the trophoblast–endometrial dialogue that stimulates early placental development. Hormones from the trophoblast, including human chorionic gonadotropin (hCG) and human placental lactogen (hPL) along with prolactin (PRL) secreted by the decidual cells (DC) stimulate the gland epithelial cells (GE) to upregulate and release nutrients, including glucose and lipid droplets (yellow), glycoproteins, such as glycodelin and osteopontin, and growth factors, including epidermal growth factor, that feed back on the trophoblast and promote proliferation (+). Rapid proliferation is essential to generate the cytotrophoblastic shell (CS) that encapsulates the conceptus and from which the endovascular aggregates that occlude the spiral arteries (SA) in the first weeks of gestation are derived. Adapted from [88] with permission.

Two key features of this new concept are that development of the placenta is in advance of, and independent of, that of the embryo. This is essential if the former is to support the latter; the early creation of a large surface area of placental villi enabling trophoblastic uptake of glucose and maternal glycoproteins that can be used in anabolic pathways by the embryo. This independence is evidenced by cases of hydatidiform mole, where large masses of placental vesicles are generated in the absence of an embryo or just rudimentary fragments [90]. The concept proposes a two-way feed-forward signalling dialogue between the trophoblast and the endometrial glands (figure 4). In the sheep, this has been referred to as a servomechanism, and sequential exposure of the gland epithelial cells to oestrogen, progesterone, interferon tau, placental lactogen and placental growth hormone promotes the secretion of growth factors and glycoproteins [91]. These hormones act on prolactin and growth hormone receptors to activate the STAT5 pathway.

The same receptors are present on the gland epithelial cells in the human and so we have proposed that an equivalent mechanism operates in our species, with the exception that hCG is the molecule of maternal recognition of pregnancy rather than interferon tau as in the sheep [92]. The Arias-Stella reaction and change in glycosylation profile of the secretions indicate that gland function is altered during early pregnancy, consistent with this concept. More compelling evidence that the mechanism occurs in the human comes from the endometrial organoids. These demonstrate upregulation of histotroph protein expression and secretion following stimulation with early pregnancy hormones, in particular prolactin [62,93]. In future, co-culture of endometrial and trophoblast organoids may provide more details of the signalling pathways involved, and the effects of the histotroph on trophoblast behaviour.

## 7. Implications of the new paradigm

**Figure 5.**
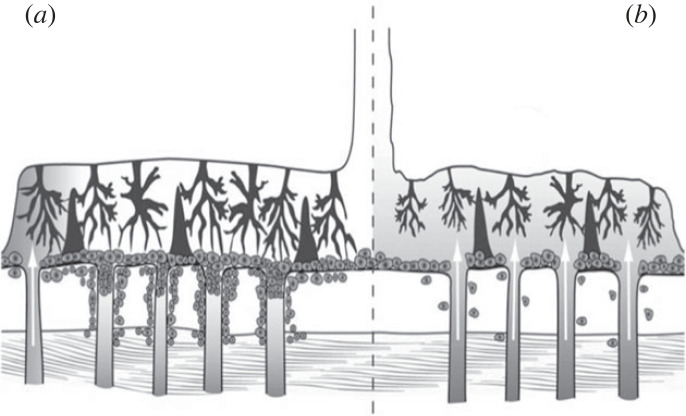
Schematic of the maternal–fetal interface in (*a*) normal pregnancies and (*b*) cases of spontaneous miscarriage. In a normal pregnancy, aggregates of endovascular trophoblast derived from a well-developed cytotrophoblastic shell occlude the lumens of the maternal spiral arteries and prevent inflow of arterial blood until 10–12 weeks of pregnancy. Some inflow may occur at the periphery of the placenta where the aggregates are less extensive, causing locally high levels of oxidative stress (shading). In cases of miscarriage, the shell is deficient, and there is widespread and early onset of the arterial circulation causing overwhelming placental oxidative stress and damage to the villous trees. From [96] with permission.

Studies have consistently reported that 12–15% of clinical pregnancies fail during the first trimester, and an even higher percentage of conceptions are thought to be lost between implantation and clinical detection of a pregnancy [94]. Between 50% and 60% of these early losses are associated with chromosomal abnormalities [95], and although there are known medical causes the majority of pregnancy failures that are chromosomally normal remain unexplained [94]. Histological examination of complete gestational sacs revealed that trophoblast proliferation and formation of the cytotrophoblastic shell are deficient in the majority of spontaneous miscarriages (figure 5) [89]. When the shell was thin and discontinuous maternal blood and clots were observed within the intervillous space, indicative of incomplete plugging of the spiral arteries. Total placental volume is also smaller and increases at a slower rate during the first trimester in pregnancies that go on to miscarry compared to healthy controls [97]. Both sets of findings indicate that placental development is compromised in cases of miscarriage, reflecting perhaps that the supply of histotroph has been inadequate or that the trophoblast has failed to respond appropriately to its stimulus. Pilot studies indicating downregulation of glycodelin, one of the principal components of histotroph, within the decidua in cases of miscarriage suggest that the former may be the case [98]. In addition, the incidence of early pregnancy failure in cases of tubal ectopic implantation, where the gestational sac develops away from the uterine glands, was found to be over 90%, supporting the essential role of histotroph in early embryo–placental development [99].

**Figure 6.**
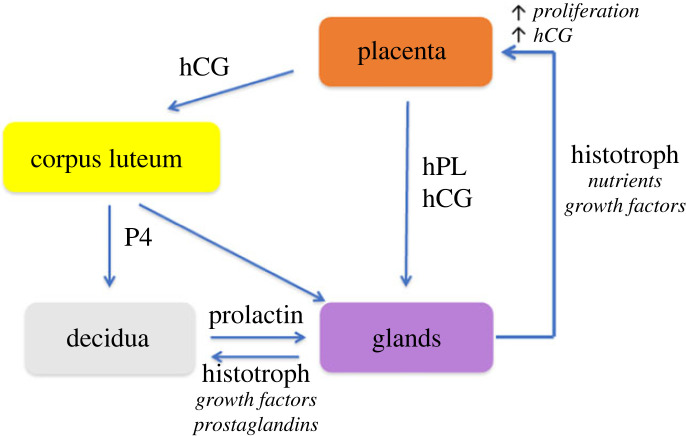
Schematic showing how decidualization is involved in the placenta–endometrial signalling dialogue in the human. Human chorionic gonadotropin (hCG) secreted by the trophoblast maintains the corpus luteum during early pregnancy. Progesterone (P4) secreted by the corpus luteum stimulates the decidual transformation in the endometrium. The decidual cells secrete prolactin, which along with P4, hCG and human placental lactogen (hPL) from the trophoblast promotes secretion of histotroph. The histotroph then feeds forward on the trophoblast, stimulating proliferation, differentiation and secretion of hCG and hPL. Growth factors and prostaglandins in the histotroph secreted baso-laterally may also reinforce decidualization. Adapted from [87] with permission.

Failure to secrete histotroph may be due to deficient stimulation of the glands or gland dysfunction. An important difference between the endometrial–trophoblast dialogue that stimulates the release of histotroph in the sheep and human is that in the human prolactin is secreted by the decidual cells and not by the trophoblast [100]. This brings the process of decidualization into the signalling loop (figure 6). Decidualization is initiated during the late secretory phase of the non-pregnant cycle, but becomes more extensive during early pregnancy when it radiates out from the implantation site to involve the entire endometrial lining of the uterus. The transformation has long been known to be stimulated by progesterone released from the corpus luteum, but emerging evidence indicates that growth factors and prostaglandins secreted vectorially from the baso-lateral aspects of the gland cells may also play a role [55,101]. Decidual cells are highly secretory and release a number of factors, including prolactin and insulin-like growth factor binding protein (IGFBP-1). Although the amino acid sequence of decidual prolactin is identical to that of pituitary prolactin, the transcriptional regulation is distinct and relies on unique retroviral promotors [102,103]. Paracrine signalling from the decidua is therefore likely to be of key importance for the secretion of histotroph during early pregnancy. A pilot study has shown evidence that expression of endometrial prolactin is downregulated in cases of early pregnancy loss [104]. The creation of endometrial assembloids, in which gland organoids have been surrounded by stromal cells that can be decidualized [105], opens new possibilities to test these hypotheses experimentally.

Miscarriage is but one of a number of complications of pregnancy that share a common association with deficient trophoblast proliferation and invasion [106]. Miscarriage is obviously the most severe, followed by pre-eclampsia and with late-onset growth restriction and pre-term delivery being at the opposite end of the spectrum. Interestingly, a recent analysis of placental chorionic villous samples from women who went on to develop pre-eclampsia or normal pregnancies identified a number of differentially expressed genes associated with decidual function, including prolactin, glycodelin and IGFBP-1, rather than trophoblast function [107,108]. The concept of dysregulated decidualization being an antecedent to complications of pregnancy was reinforced by experiments in which stromal cells isolated from midsecretory endometrial biopsies obtained from women who had suffered pre-eclampsia in the previous 1–5 years were decidualized *in vitro* [109,110]. The panel of differentially expressed genes showed considerable overlap with those identified from the chorionic villous samples.

Much attention has been paid to the development of complications during pregnancy, but these findings support the idea that their pathophysiology may start prior to pregnancy and lie in defective decidualization. A spectrum of impairment matching the severity of the complications has recently been proposed [111]. Aberrant decidualization may affect many aspects of decidual function during pregnancy, including interactions between the trophoblast and maternal immune cells, trophoblast invasion, spiral artery remodelling, as well as secretion of histotroph. Glycodelin within the histotroph has been shown to mediate many of these effects *in vitro* [112], and is present within the extracellular matrix of the superficial endometrium due to breaks in the epithelial lining of the glands [93]. Some actions of glycodelin are dependent on its pattern of glycosylation [113,114], and so are potentially susceptible to endoplasmic reticulum stress and other stresses within the gland epithelial cells that may disrupt glycosylation. The same may apply to other maternal glycoproteins in the histotroph, but no data are available as yet. Further research is obviously required, but if this new paradigm is confirmed then ensuring endometrial function is optimal prior to conception should be given greater health care priority. The ability to derive physiologically responsive organoids that faithfully replicate the transcriptome of the *in vivo* endometrium non-invasively from menstrual flow opens the possibility to screen gland function in women pre-conceptionally [115]. Possible therapeutic interventions could be tested on the organoids in a personalized approach to reduce cell stress, cytokine imbalance or supplement gland stimulation.

It is fully recognized that this approach will not prevent all complications of pregnancy, but as attempts to create effective therapies once the complications are established have failed to date it is perhaps worth changing strategy and concentrating on their prevention. Understanding the basic physiology underpinning placental development is key, and this new paradigm opens novel avenues of the maternal–fetal cross-talk to explore.

## Data Availability

This article has no additional data.
